# The nonlinear relationship between the ratio of non-high-density lipoprotein cholesterol to high-density lipoprotein cholesterol and the risk of diabetic kidney disease in patients with type 2 diabetes mellitus

**DOI:** 10.3389/fmed.2025.1492483

**Published:** 2025-02-19

**Authors:** Dan-Xuan Cai, Ye-Hong Huang, Ni-Na Lin, Yun-Feng Zhang, Shu-Qin Huang, Yun Han, Xin-Yu Hu, Song-Tao Cai, Yan-Ling Tao

**Affiliations:** ^1^Shenzhen Clinical Medical College, Guangzhou University of Chinese Medicine, Shenzhen, China; ^2^Department of Nursing, Longgang Central Hospital of Shenzhen, Shenzhen, China; ^3^Department of Health Management, The Second Affiliated Hospital, School of Medicine, The Chinese University of Hong Kong, Shenzhen & Longgang District People’s Hospital of Shenzhen, Shenzhen, China; ^4^Department of Endocrinology, The Second Affiliated Hospital, School of Medicine, The Chinese University of Hong Kong, Shenzhen & Longgang District People’s Hospital of Shenzhen, Shenzhen, China; ^5^Department of Science and education, The Second Affiliated Hospital, School of Medicine, The Chinese University of Hong Kong, Shenzhen & Longgang District People’s Hospital of Shenzhen, Shenzhen, China; ^6^Hanlin Cheng Community Health Service Center, The Second Affiliated Hospital, School of Medicine, The Chinese University of Hong Kong, Shenzhen & Longgang District People’s Hospital of Shenzhen, Shenzhen, China

**Keywords:** database research, diabetic kidney disease, NHANES, NHHR, type 2 diabetes mellitus

## Abstract

**Background:**

The ratio of non-high-density lipoprotein cholesterol to high-density lipoprotein cholesterol (NHHR) is a novel marker related to atherosclerosis, but its role in diabetic kidney disease (DKD) remains unclear. This study investigated the relationship between NHHR and DKD risk in patients with type 2 diabetes mellitus (T2DM) and evaluated its potential as a marker for early DKD screening.

**Methods:**

Data from adults with T2DM participating in the National Health and Nutrition Examination Surveys (NHANES) from 1999 to 2018 were analyzed. Demographic information, laboratory tests, and other relevant information were collected. To evaluate the correlation between NHHR levels and DKD risk, weighted multivariable logistic regression and weighted restricted cubic spline (RCS) analyses were employed. Furthermore, threshold effect analysis was employed to further explore the relationship at different NHHR levels, and subgroup analyses validated the results.

**Results:**

The study enrolled a total of 3,243 participants, comprising 1,258 individuals with DKD (38.79%) and 1,985 individuals without DKD (61.21%). The multivariable logistic regression analysis showed that T2DM patients with higher NHHR levels exhibited a 45% reduction in the risk of developing DKD in comparison to those with lower NHHR levels (Q2 vs. Q1: OR 0.55, 95% CI 0.40–0.76). The weighted RCS analysis revealed a nonlinear correlation between NHHR and the risk of DKD in patients with T2DM (*P* for nonlinear = 0.003), with the RCS plot exhibiting an L-shaped association. A negative association was observed between NHHR levels and the risk of DKD when NHHR was ≤2.82 (OR 0.63, 95% CI 0.49–0.83). A statistically significant correlation between NHHR and DKD risk was not observed when NHHR was >2.82. The subgroup analyses indicated that age may have an interaction effect on this association at higher NHHR levels (*p* for interaction<0.05).

**Conclusion:**

Our findings revealed a non-linear relationship between the NHHR levels and the risk of DKD in adult T2DM patients in the United States. Managing the NHHR levels in the right range in T2DM patients can help reduce the risk of DKD. This suggests that NHHR may be a valuable and easily measurable biomarker for identifying those at risk for DKD, thereby promoting early intervention and improved disease management.

## Introduction

Diabetic kidney disease (DKD) is one of the primary long-term complications of diabetes mellitus, characterized by proteinuria and progressive renal failure (1, 2). The global prevalence of diabetes continues to rise, with estimates suggesting that by 2021 approximately 537 million adults worldwide were affected by diabetes, and this figure is expected to reach 783 million by 2045 (3). Research has identified DKD as the leading cause of chronic kidney disease (CKD) and end-stage renal disease requiring dialysis or transplantation globally (4). About 30 to 40% of individuals with diabetes develop DKD, and the prevalence of DKD continues to rise (5). DKD contributes to a significant portion of the disease burden globally and poses a substantial socio-economic and healthcare security challenge (4). Therefore, early intervention in diabetic patients to reduce the risk of DKD is crucial.

Extensive evidence has demonstrated that dyslipidemia is a key factor in the progression of kidney disease in diabetic patients (6, 7). Dyslipidemia is known to contribute to renal damage by activating TGF-*β*, leading to the production of reactive oxygen species and subsequent harm to the glomerulus and its glycocalyx (8). A meta-analysis of 20 cohorts demonstrated that low levels of high-density lipoprotein cholesterol (HDL-C) are a significant risk factor for DKD, with each 1 mmol/L increase in HDL-C associated with a 22% reduction in DKD risk (9). A cross-sectional study of 3,698 Chinese participants found that elevated triglyceride levels were strongly associated with an increased risk of DKD, with each unit increase in triglycerides raising the risk by 16%, while non-high-density lipoprotein cholesterol (non-HDL-C) was not significantly associated with DKD risk (10). Although the relationship between HDL-C as well as non-HDL-C and DKD has been discussed in the previous literature, these studies have focused on individual lipid indices and have not fully explored the potential value of other composite lipid indices.

The ratio of non-high-density lipoprotein cholesterol to high-density lipoprotein cholesterol (NHHR) is a novel and prospective composite lipid marker associated with atherosclerosis, which is calculated from the ratio of non-HDL-C to HDL-C. Several previous studies have shown that the NHHR is significantly associated with diabetes mellitus (11), non-alcoholic fatty liver disease (12), insulin resistance (13), kidney stones (14), and carotid plaque (15, 16). A cohort study found that NHHR has better accuracy than other conventional lipid parameters (HDL-C, TC and non-HDL-C) in predicting the risk of diabetes-related disease (AUC = 0.7405, 95%CI 0.7158–0.7651) (11). NHHR gives a more comprehensive picture of the overall lipid metabolism of the human body compared to other single indicators (17). Considering the extensive impact of diabetes on human metabolism, it is of great interest to further investigate the value of NHHR in predicting the risk of diabetes-related diseases. However, there is still a gap in research on the link between NHHR and DKD risk.

Based on this, the present study conducted a cross-sectional survey of T2DM patients using the NHANES database. The aim was to analyze the complex relationship between NHHR and the risk of DKD, to provide new clinical indicators for early screening, and thus to help improve the prevention and management of DKD.

## Materials and methods

### Study population

This research utilized data sourced from the NHANES database. NHANES collected comprehensive health, nutritional, and sociological information from various ethnic groups in the United States. To ensure the sample accurately reflected the broader population, NHANES employed a complex multi-stage probability sampling methodology. The National Centre for Health Statistics (NCHS) Research Ethics Review Board reviewed and approved the design of NHANES. Written informed consent was obtained from each participant before they joined the study. The data used in this study, along with further details about NHANES, are available at https://www.cdc.gov/nchs/nhanes/.

**Figure 1:**
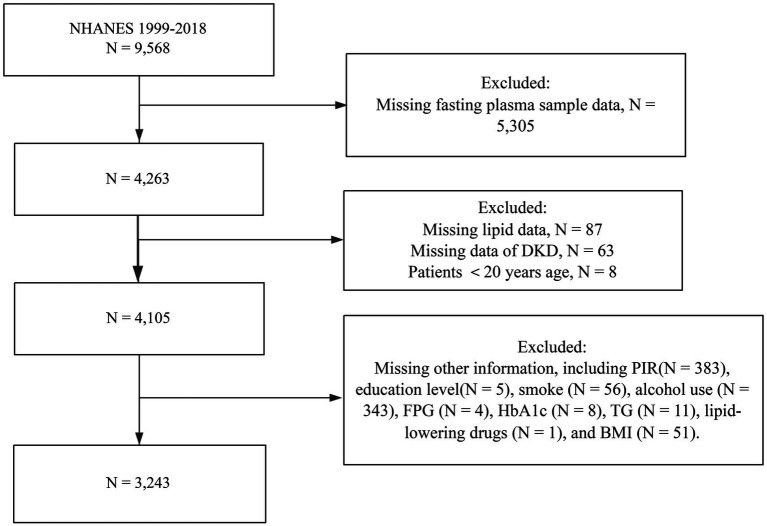
Flowchart for inclusion of subjects.

We selected T2DM participants from the NHANES database between 1999 and 2018. A total of 9,568 patients with T2DM were initially included, and after screening out patients with no fasting blood test data (*n* = 5,305), patients with missing NHHR data (*n* = 87), patients with missing information related to DKD (*n* = 63), and patients <20 years of age (*n* = 8), there were a total of 4,105 cases. Then, after excluding participants with missing data on covariates such as poverty income ratio (PIR) (*n* = 383), education level (*n* = 5), smoke (*n* = 56), alcohol use (*n* = 343), fasting plasma glucose (FPG) (*n* = 4), glycated hemoglobin (HbA1c) (*n* = 8), triglyceride (TG) (*n* = 11), lipid-lowering drugs (*n* = 1), and body mass index (BMI) (*n* = 51), 3,243 patients were finally included in the study. A comprehensive flowchart illustrating the inclusion and exclusion criteria is presented in Figure 1.

### Exposure and outcome definitions

NHHR was the exposure variable in this study, defined as the difference between total cholesterol and HDL-C over HDL-C (16). Fasting blood specimens were collected from the participants by the staff for enzymatic lipid determination using an automated biochemical analyzer. Total cholesterol levels were assessed using the Roche Cobas 6000 chemistry analyzer and the Roche Modular P system.

DKD was the outcome variable in this study. The diagnosis of DKD in this study was defined according to the CKD guidelines (18): patients with T2DM who had a urinary albumin-to-creatinine ratio (UACR) >30 mg/g and/or an estimated glomerular filtration rate (eGFR) <60 mL/min/1.73 m^2^ were diagnosed with DKD. The diagnostic criteria for diabetes mellitus in this study were based on the following (19): (1) having been diagnosed with diabetes mellitus by a physician; (2) glycosylated hemoglobin≥6.5%; (3) fasting blood glucose≥7.0 mmol/L; (4) random blood glucose≥11.1 mmol/L; (5) OGTT≥11.1 mmol/L; and (6) use of antidiabetic medication or insulin. The eGFR was derived using the CKD-EPI creatinine equation (20), which incorporates factors such as age, gender, race, and serum creatinine. The UACR was determined by calculating the ratio of urinary albumin to creatinine.

### Covariate definitions

Based on previous relevant studies and clinical experience, we collected a number of covariate data that could have influenced the results (18, 21, 22). Demographic covariates included sex (male/female), age, race, education level (below high school/high school/above high school), and PIR. Lifestyle-related covariates included smoke (yes/no), alcohol use (yes/no), physical activity (no/moderate/vigorous), and BMI. Covariates related to underlying disease history included hypertension, cardiovascular disease (CVD), and lipid-lowering drugs. Laboratory test-related covariates included HbA1c, FPG, alanine aminotransferase (ALT), aspartate aminotransferase (AST), eGFR, creatinine (Cr), uric acid (UA), blood urea nitrogen (BUN), urinary albumin (Ualb), urine creatinine (Ucr), UACR, TG, total cholesterol (TC), HDL-C and low-density lipoprotein cholesterol (LDL-C).

The criteria used to diagnose hypertension in this research are defined as follows (23): (1) previously diagnosed with hypertension by a physician; (2) an abnormal average blood pressure; and (3) the use of anti-hypertensive medication. CVD is comprised of coronary heart disease, congestive heart failure, stroke, angina, and myocardial infarction, as identified through the Medical Conditions Questionnaire (MCQ). Comprehensive methods for collecting all covariates can be found on the NHANES website.

### Statistical analysis

Due to the complex, stratified sampling methodology used in the collection of the NHANES database, all statistical analyses in this study used sample weights according to NHANES recommendations. For continuous variables, data were presented as mean values with standard errors, and inter-group differences were assessed using weighted one-way ANOVA. Categorical variables were represented as percentages with 95% confidence intervals (CIs), and differences across groups were evaluated via weighted chi-square tests. The association between NHHR and DKD was analyzed using the weighted multivariable logistic regression model. In accordance with the STROBE (Strengthening the Reporting of Observational Studies in Epidemiology) guidelines, four models were constructed: Model 1 without covariate adjustments, Models 2 and 3 with incremental covariate adjustments, and Model 4, the fully adjusted model, which accounted for age, sex, race, PIR, education level, smoke, alcohol use, physical activity, BMI, hypertension, CVD, lipid-lowering drugs, FBG, HbA1c, ALT, AST, Cr, UA, BUN, and TG.

We then analyzed the non-linear association among NHHR and DKD using the weighted RCS model. After fully adjusting for the covariates of interest, if the association exhibits non-linearity, the threshold probability is estimated and the association on either side of the threshold is analyzed using the threshold effects analysis model.

We finally performed multiple subgroup analyses to test the stability of the outcome. These subgroup analyses were stratified by age (<60/≥60), sex (male/female), race (Mexican American/Non-Hispanic Black/Non-Hispanic White/Other Hispanic/Other Race), HbA1c (<7/≥7), BMI (<25/≥25, <30/≥30), smoke (yes/no), hypertension (yes/no), and CVD (yes/no). All statistical analyses were carried out using R software, version 4.3.2. The differences were considered to be statistically significant at *p* < 0.05 (two-sided).

## Results

### Baseline characteristics of participants

A total of 3,243 eligible T2DM patients were involved in this research from NHANES 1999–2018. After weighted processing, these 3,243 subjects represent approximately 24.98 million non-institutionalized citizen population in the United States. Supplementary Table 1 demonstrates the weighted baseline characteristics of the included T2DM patients. The average age of the participants was 58.80 ± 0.33 years. A total of 1,710 males (51.5%) and 1,533 females (48.5%) were included in this study with a DKD composition ratio of 38.79%. The analysis revealed a statistically significant difference between patients with and without DKD across various factors, including age, education level, alcohol use, hypertension, CVD, physical activity, lipid-lowering drugs, PIR, HbA1c, FPG, ALT, AST, eGFR, Cr, UA, BUN, urine albumin, Ucr, UACR, TG, and LDL-C (*p* < 0.05).

### Baseline characteristics of T2DM patients grouped according to NHHR quartiles

Table 1 presents the weighted baseline characteristics of subjects, grouped based on the NHHR quartiles. The mean NHHR of the participants being 3.19 ± 0.04, and the interquartile range of NHHR from 1 to 4 was 0.31–2.08, 2.08–2.87, 2.87–3.89, and 3.89–26.67, respectively. Between-group differences in NHHR quartiles were observed between the variables of age, sex, race, hypertension, DKD, physical activity, lipid-lowering drugs, PIR, BMI, HbA1c, FPG, ALT, AST, eGFR, UA, BUN, Ucr, TG, TC, HDL-C, and LDL-C (*p* < 0.05).

**Table 1:** The weighted baseline characteristics of the study population by the quartiles of NHHR from NHANES 1999–2018.

Characteristic	Quartiles of NHHR				*p*-value
	Q1 (0.31–2.08)	Q2 (2.08–2.87)	Q3 (2.87–3.89)	Q4 (3.89–26.67)	
Age, years	63.11 ± 0.69	60.26 ± 0.58	58.12 ± 0.61	54.22 ± 0.66	<0.0001
Sex (%)					<0.0001
Male	367 (43.18)	392 (45.41)	447 (54.66)	504 (61.78)	
Female	444 (56.82)	419 (54.59)	363 (45.34)	307 (38.22)	
Race (%)					<0.0001
Mexican American	118 (6.81)	138 (6.84)	177 (9.45)	214 (12.08)	
Non-Hispanic Black	243 (18.00)	195 (14.08)	160 (11.97)	115 (8.94)	
Non-Hispanic White	307 (62.55)	354 (68.01)	323 (65.54)	334 (66.03)	
Other Hispanic	63 (4.12)	66 (5.02)	89 (6.60)	90 (6.50)	
Other Race	80 (8.51)	58 (6.05)	61 (6.44)	58 (6.45)	
Education levels (%)					0.172
Below high school	114 (6.68)	133 (9.63)	145 (9.97)	166 (11.29)	
High school	324 (40.13)	328 (40.16)	347 (42.64)	335 (41.69)	
Above high school	373 (53.19)	350 (50.21)	318 (47.39)	310 (47.01)	
Smoke (%)					0.276
No	425 (51.53)	424 (51.36)	412 (48.33)	355 (45.70)	
Yes	386 (48.47)	387 (48.64)	398 (51.67)	456 (54.30)	
Alcohol use (%)					0.679
No	137 (15.44)	159 (17.21)	152 (15.71)	121 (14.52)	
Yes	674 (84.56)	652 (82.79)	658 (84.28)	690 (85.48)	
Hypertension (%)					0.019
No	209 (29.74)	221 (28.73)	236 (29.11)	284 (37.01)	
Yes	602 (70.26)	590 (71.27)	574 (70.89)	527 (62.99)	
Cardiovascular disease (%)					0.206
No	578 (74.19)	623 (78.67)	634 (78.53)	646 (80.14)	
Yes	233 (25.80)	188 (21.33)	176 (21.47)	165 (19.86)	
Diabetic kidney disease (%)					0.012
No	480 (60.01)	524 (70.04)	482 (63.05)	499 (66.40)	
Yes	331 (39.99)	287 (29.96)	328 (36.95)	312 (33.60)	
Physical activity (%)					0.014
No	523 (59.99)	514 (56.35)	480 (56.68)	464 (49.76)	
Moderate	194 (26.23)	180 (28.16)	201 (26.22)	201 (27.18)	
Vigorous	94 (13.77)	117 (15.49)	129 (17.10)	146 (23.05)	
Lipid-lowering drugs (%)					<0.0001
No	292 (35.20)	398 (45.62)	508 (61.72)	587 (71.72)	
Yes	519 (64.80)	413 (54.38)	302 (38.28)	224 (28.28)	
PIR	2.87 ± 0.09	2.87 ± 0.08	2.70 ± 0.09	2.57 ± 0.07	0.012
BMI, kg/m^2^	30.24 ± 0.34	32.95 ± 0.40	33.50 ± 0.39	33.70 ± 0.37	<0.0001
HbA1c, %	6.69 ± 0.06	6.85 ± 0.07	6.95 ± 0.07	7.36 ± 0.08	<0.0001
FPG, mg/dL	135.87 ± 2.06	145.91 ± 2.63	147.40 ± 2.51	164.31 ± 2.78	<0.0001
ALT, IU/L	24.70 ± 0.66	25.75 ± 0.67	28.76 ± 0.79	33.06 ± 1.10	<0.0001
AST, IU/L	26.07 ± 0.63	25.19 ± 0.51	25.92 ± 0.57	28.45 ± 0.93	0.029
eGFR, ml/min/1.73m^2^	79.94 ± 1.11	83.60 ± 1.06	86.00 ± 0.91	89.90 ± 1.13	<0.0001
Cr, mg/dL	1.03 ± 0.04	0.94 ± 0.02	0.94 ± 0.02	0.92 ± 0.02	0.100
UA, mg/dL	5.54 ± 0.07	5.76 ± 0.06	6.02 ± 0.07	6.08 ± 0.08	<0.0001
BUN, mg/dL	16.45 ± 0.37	15.98 ± 0.28	15.62 ± 0.27	15.13 ± 0.30	0.037
Ualb, mg/L	84.31 ± 11.73	106.36 ± 24.29	122.12 ± 22.37	188.09 ± 40.11	0.089
Ucr, mg/dL	115.34 ± 3.35	119.69 ± 3.43	129.65 ± 3.97	133.50 ± 3.17	<0.001
UACR, mg/g	105.74 ± 21.79	103.71 ± 21.71	98.56 ± 16.21	166.38 ± 33.03	0.303
TG, mg/dL	93.93 ± 1.91	129.39 ± 3.04	168.40 ± 3.37	294.04 ± 13.50	<0.0001
TC, mg/dL	163.50 ± 1.78	176.71 ± 1.98	193.19 ± 1.91	225.90 ± 2.04	<0.0001
HDL-C, mg/dL	64.33 ± 1.07	51.12 ± 0.54	44.54 ± 0.44	37.34 ± 0.37	<0.0001
LDL-C, mg/dL	80.38 ± 1.06	100.17 ± 1.44	115.85 ± 1.74	140.16 ± 1.90	<0.0001
NHHR	1.60 ± 0.01	2.46 ± 0.01	3.35 ± 0.01	5.18 ± 0.06	<0.0001

Continuous variables were presented as Mean ± SE, *p*-value was calculated by survey-weighted linear regression. Categorical variables were presented as the percentage (95% confidence interval), *p*-value was calculated by the survey-weighted Chi-square test.

NHANES, National Health and Nutrition Examination Survey; PIR, poverty income ratio; BMI, body mass index; HbA1c, glycated hemoglobin; FPG, fasting plasma glucose; ALT, alanine aminotransferase; AST, aspartate aminotransferase; eGFR, estimated glomerular filtration rate; Cr, creatinine; UA, uric acid; BUN, blood urea nitrogen; Ualb, urinary albumin; Ucr, urine creatinine; UACR, urinary albumin/creatinine ratio; TG, triglyceride; TC, total cholesterol; HDL-C, high-density lipoprotein cholesterol; LDL-C, low-density lipoprotein cholesterol; NHHR, non-high-density lipoprotein cholesterol to high-density lipoprotein cholesterol ratio.

### The association between NHHR and DKD risk

The linear relationship between NHHR and DKD risk is shown in Table 2. Our results show that when NHHR is analyzed as a continuous variable, it is not linearly related to the risk of DKD, either in the Model 1 (OR 0.98, 95%CI 0.92–1.04) or in the Model 4 (OR 0.90, 95%CI 0.81–1.00). We divided the NHHR into quarters for analysis. Model 1 is a crude model with no adjustment for any covariates. In Model 1, the Q2 exhibited a lower risk of DKD when compared to the lowest NHHR quartile (OR 0.64, 95%CI 0.49–0.84). After adjusting for demographic characteristics and lifestyle covariates, the risk of DKD for Q2 in Models 2 and 3 is still lower than the lower NHHR quartile. After controlling for various covariates including age, sex, race, PIR, education level, smoke, alcohol use, physical activity, BMI, hypertension, CVD, lipid-lowering drugs, FBG, HbA1c, ALT, AST, Cr, UA, BUN, and TG, this relationship remains stable in Model 4, with a 45% reduction in the risk of DKD in Q2 compared to Q1 (OR 0.55, 95%CI 0.40–0.76). Model 4 is the fully adjusted model that takes into account all covariates and provides the most realistic reflection of the relationship between NHHR and DKD risk. The test for trend showed a statistically significant interquartile regression trend in model 4 (*p* < 0.05), suggesting that changes in different NHHR quartiles were strongly associated with the risk of DKD.

**Table 2:** The relation between NHHR and DKD risk.

	OR (95%CI) *p*-value			
	Model 1	Model 2	Model 3	Model 4
NHHR (continuous)	0.98 (0.92, 1.04) 0.415	1.04 (0.98, 1.11) 0.223	1.02 (0.95, 1.09) 0.574	0.90 (0.81, 1.00) 0.062
NHHR (quartiles)				
Q1	ref	ref	ref	ref
Q2	0.64 (0.49, 0.84) 0.001	0.70 (0.53, 0.91) 0.009	0.65 (0.50, 0.86) 0.003	0.55 (0.40, 0.76) <0.001
Q3	0.88 (0.66, 1.18) 0.397	1.04 (0.78, 1.40) 0.783	0.94 (0.69, 1.28) 0.686	0.78 (0.55, 1.10) 0.155
Q4	0.76 (0.58, 0.99) 0.041	1.02 (0.77, 1.36) 0.886	0.93 (0.69, 1.26) 0.648	0.57 (0.40, 0.82) 0.003
*P* for trend	0.274	0.300	0.697	0.038

Model 1: no covariates were adjusted.

Model 2: adjusted for age, sex, race, PIR, and education level.

Model 3: adjusted for age, sex, race, PIR, education level, smoke, alcohol use, physical activity, BMI, hypertension, CVD, and lipid-lowering drugs.

Model 4: adjusted for age, sex, race, PIR, education level, smoke, alcohol use, physical activity, BMI, hypertension, CVD, lipid-lowering drugs, FBG, HbA1c, ALT, AST, Cr, UA, BUN, and TG.

### The nonlinear relationship between NHHR and DKD risk

**Figure 2:**
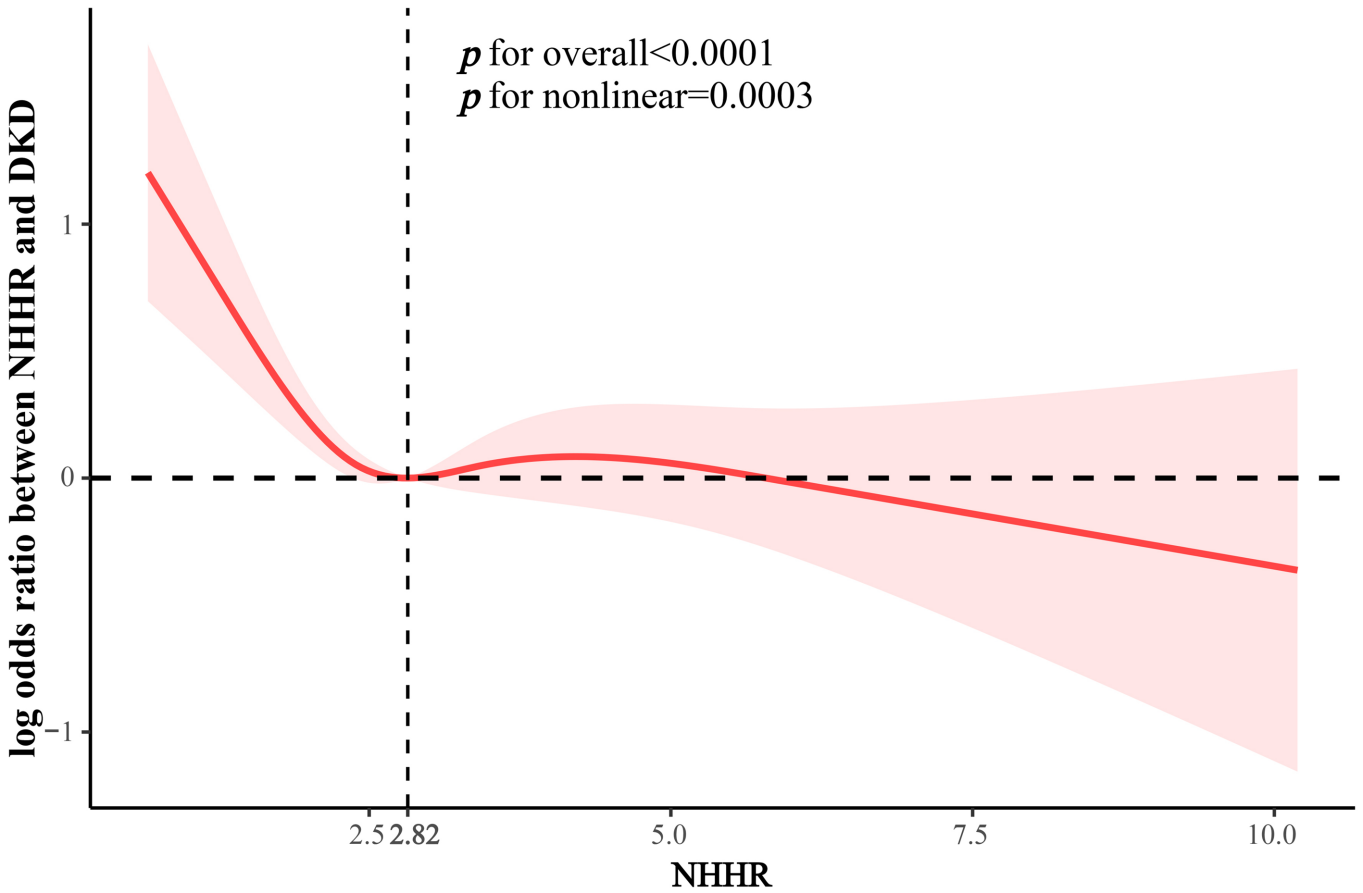
The nonlinear association between NHHR and DKD risk.

To further investigate the nonlinear correlation between NHHR and DKD, we performed the weighted RCS analysis. The results showed that NHHR was nonlinearly correlated with the risk of developing DKD after adjusting for covariates such as age, sex, race, PIR, education level, smoke, alcohol use, physical activity, BMI, hypertension, CVD, lipid-lowering drugs, FBG, HbA1c, ALT, AST, Cr, UA, BUN, and TG (*P* for nonlinear = 0.003). The graph of the relationship between NHHR and the risk of DKD is demonstrated in Figure 2, which shows an L-shaped relationship between NHHR and the risk of DKD. When the NHHR is less than 2.82, the risk of DKD decreases with increasing NHHR, whereas when the NHHR is greater than 2.82, the decrease in DKD risk leveling off as the NHHR continued to increase. To further explore this L-shaped relationship, we further analyzed the relationship on either side of the NHHR threshold using two linear regression models. As shown in Table 3, the risk of DKD in T2DM patients was reduced by 37% for each unit increase in NHHR when NHHR was ≤2.82 (OR 0.63, 95%CI 0.49–0.83). No significant association was observed between changes in NHHR and DKD risk when NHHR >2.82. Interaction tests suggested an interaction effect of different NHHR ranges on this relationship (*p* for interaction<0.05). Our results suggested an L-shaped relationship between NHHR and DKD risk. When NHHR is controlled around 2.82, the risk of DKD is lower in patients with T2DM, which provided a reference for lipid management in clinical treatment of diabetes.

**Table 3:** Threshold effect analysis of the relationship between NHHR and DKD risk.

	OR (95%CI)	*p*-value	*p* for interaction
NHHR ≤ 2.82	0.63 (0.49, 0.83)	0.001	0.001
NHHR > 2.82	1.04 (0.93, 1.15)	0.490	

Adjusted for age, sex, race, PIR, education level, smoke, alcohol use, physical activity, BMI, hypertension, CVD, lipid-lowering drugs, FBG, HbA1c, ALT, AST, Cr, UA, BUN, and TG.

### Subgroup analysis

**Figure 3:**
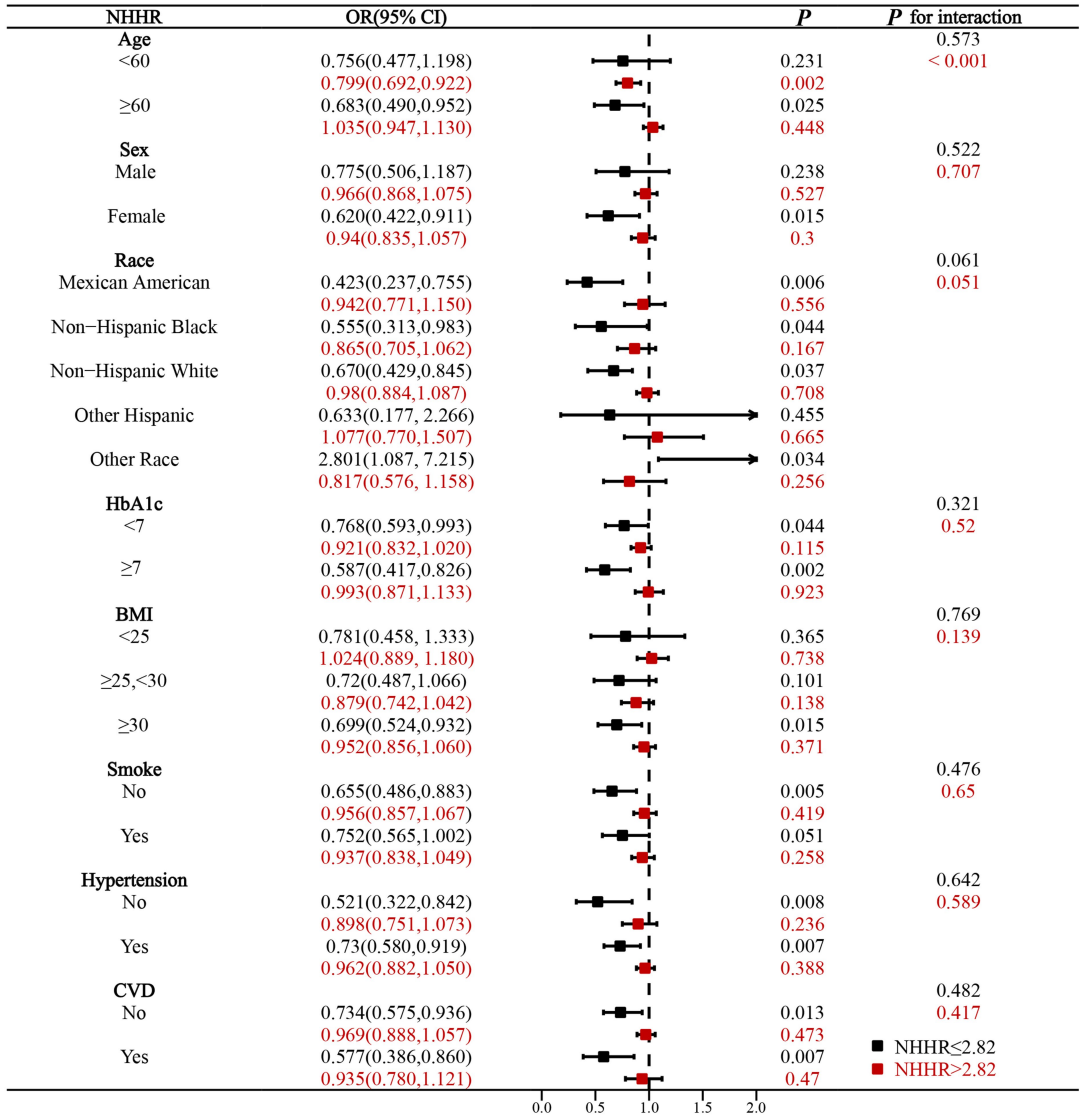
Forest plot of subgroup analysis of NHHR with DKD risk.

To further explore the relationships found previously, we conducted multiple subgroup analyses. The analysis was stratified according to several factors: age, sex, race, HbA1c, BMI, smoke, hypertension, and CVD, and the forest plots for multiple subgroup analyses are shown in Figure 3. The results of the subgroup analyses are displayed in the Supplementary Table 2. When NHHR≤2.82, there was no statistically significant interaction test between multiple factors, which means that these multiple factors did not influence this association (*p* for interaction>0.05). When NHHR >2.82, interaction tests indicated that age had a significant effect on the relationship between NHHR and DKD risk (*p* for interaction<0.05). For the T2DM population aged <60 years, NHHR was negatively related to the risk of DKD (OR 0.80, 95%CI 0.69–0.92). For T2DM populations aged≥60 years or older, NHHR was positively associated with DKD risk, but this association was not statistically significant (OR 1.03, 95%CI 0.95–1.13). Notably, the results showed that NHHR exhibited a significant risk reduction for DKD risk in females, Mexican Americans, non-Hispanics, those with HbA1c ≥ 7, BMI ≥ 30, non-smokers, and those without hypertension when NHHR was ≤2.82. This suggested that in the clinical management of diabetes, strict lipid management according to the NHHR for these populations might provide additional long-term benefits, leading to a significant reduction in the risk of DKD. Subgroup analyses of multiple factors demonstrated the robustness of the relationship between DKD risk and NHHR.

## Discussion

This study is the first to explore the link between NHHR and DKD risk in T2DM patients. Our analysis based on a large sample from the United States, revealed a nonlinear relationship between NHHR and DKD risk in T2DM patients, with age influencing this association when NHHR >2.82. We found that NHHR was negatively related to the risk of DKD when the NHHR was within 2.82 and that the risk of DKD was lowest when the NHHR was controlled at around 2.82. The protective effect of NHHR against DKD was more evident in women, non-Hispanics, individuals with poor glycaemic control (HbA1c ≥ 7), obesity (BMI ≥ 30), non-smokers, and those without hypertension at NHHR ≤2.82. This provides an accurate reference range for long-term lipid management in patients with T2DM and allows for a more precise management strategy that takes into account the patient’s specific physical characteristics (e.g., gender, age, etc.). The NHHR as a novel and promising lipid marker can quantify the role of dyslipidaemia in the risk of developing DKD.

Lipid metabolism disorders are one of the common characteristics of T2DM patients and one of the risk factors for DKD (24, 25), and previous studies have mainly focused on the two indicators of HDL-C and non-HDL-C. Several studies have demonstrated that elevated HDL-C levels correlate with a reduced risk of DKD development (26, 27). However, recent studies have found a controversial relationship between very high or very low HDL-C levels and DKD risk. A cross-sectional study based on a Chinese population found a nonlinear relationship between threshold intervals between HDL-C levels and DKD incidence, with patients with HDL-C ≤ 0.94 mmol/L or HDL-C > 1.54 mmol/L having a significantly higher risk of DKD after controlling for confounders (28). This is consistent with our results, suggesting that the effect of lipids on the risk of DKD may be nonlinear and that extremes of too high or too low values should be noted in lipid management. The non-HDL-C is an indicator that takes into account all atherogenic lipoproteins, which are also strongly linked to the development of DKD. It includes low-density lipoprotein (LDL) cholesterol, lipoprotein (a), medium-density lipoprotein (MDL), and very-low-density lipoprotein (VLDL) remnants. A real-world study based on 72,267 patients showed that each 1 mg/dL increase in non-HDL-C resulted in a 0.2% increased risk of microvascular complications in patients with T2DM (29). The NHHR is a new type of lipid index that takes into account both the protective effects of HDL-C and the risk factors of non-HDL-C to provide a more comprehensive picture of an individual’s lipid metabolism. Extensive evidence has confirmed that the NHHR is an excellent predictor of lipid-related diseases and that it better reflects the complex lipid metabolism of diabetic patients (14, 30, 31). Our results found that NHHR was nonlinearly linked to the risk of developing DKD in T2DM patients. It suggested that when NHHR is controlled below 2.82, the higher the NHHR, the lower the risk of developing DKD. To maintain high NHHR levels and reduce the risk of DKD, clinical interventions should focus on lipid optimization through statins or other lipid-lowering medications, regular physical activity, weight management, and smoking cessation (32–37). These strategies can lower non-HDL-C while increasing HDL-C, thereby improving NHHR and reducing DKD risk. This provides a clear data reference for the actual clinical management of lipid levels in T2DM patients.

The exact biological mechanism by which NHHR affects DKD risk may involve disturbance in lipid metabolism. Our findings demonstrated an L-shaped correlation between NHHR and DKD risk in patients with T2DM. When NHHR levels are low, HDL-C levels are higher, whereas non-HDL-C levels are lower. At this time, the vasculoprotective effects of HDL-C dominate, exerting renoprotective, anti-inflammatory, cholesterol efflux, antioxidant, and vascular endothelium-protective functions, thereby reducing the risk of diabetes-related microangiopathy (38–42). HDL-C has antioxidant properties that prevent oxidative stress-induced damage, which in turn prevents endothelial dysfunction, pro-inflammatory pathways in the vascular wall, and alterations of lipoproteins on lipids and proteins (43). Either HDL-C deficiency or dysfunction can impede the process of reverse cholesterol transport, which plays an important role in glomerulosclerosis and tubulointerstitial injury (44). A role for HDL-C in DKD may be important not only because diabetic patients are known to have low HDL-C but also because HDL-C function is impaired by glycosylation end-products (43–45). When the NHHR exceeds the threshold, the protective effect of HDL-C does not offset the negative effects of non-HDL-C levels. The risk of atherosclerosis and microangiopathy began to increase, resulting in the decrease in DKD risk leveling off as the NHHR continued to increase. Elevated non-HDL-C can induce oxidative stress, leading to an increase in oxygen free radicals, and these free radicals directly damage the glomerular filtration membrane, leading to proteinuria and renal failure (46). Decreased HDL-C and elevated non-HDL-C enhanced macrophage infiltration and production of excess extracellular matrix, leading to accelerated inflammation and promoting the progression of nephropathy (47). Our findings suggested that keeping the NHHR around 2.82 resulted in the greatest benefit in reducing the risk of DKD for patients with T2DM. The NHHR can be used in the clinic for early identification of patients at high risk for type 2 diabetes as an easily measured composite lipid indicator. Compared to a single lipid index, NHHR combines multiple lipid components and may provide a more comprehensive picture of an individual’s metabolic health. Therefore, it is expected to be an early screening tool for DKD and help clinicians better prevent and manage diabetes-related renal complications.

Our results suggested an interaction of age for NHHR with DKD risk when NHHR>2.82. One possible explanation is that higher NHHR may reflect early atherosclerosis as well as microvascular disease in younger populations that may be at higher risk for DKD. In the elderly population, however, the impact of high NHHR on DKD is overshadowed by the effects of a longer duration of diabetes and other chronic conditions. Aging affects lipid metabolism and renal function. As individuals age, changes in lipid metabolism occur through the modulation of key pathways related to lipid transport. These pathways include adipose tissue lipolysis, lipoprotein metabolism, triglyceride metabolism, and alterations in lipid transport proteins (48). It has been found that lipolysis in the adipose tissue of the elderly diminishes with age, associated with reduced catecholamine availability and decreased hormone-sensitive lipase activity (49–51). These changes may lead to an accumulation of body fat and an increased supply of fatty acids, which may trigger chronic inflammation and insulin resistance, factors that are strongly associated with the progression of diabetic nephropathy. Aging also leads to a decrease in the organ’s ability to utilize lipids as an energy substrate, and lipids tend to accumulate in the kidneys, particularly in the tubular and glomerular regions (52, 53). In older adults, the kidneys may be subjected to a greater lipid load, which can trigger renal lipotoxic effects and lead to an increased risk of DKD. In this case, another possible explanation is that a low NHHR may help to reduce these lipotoxic effects and provide renal protection. This is consistent with the age-related interactions found in our findings.

This is the first study utilizing a large dataset to evaluate the risk of DKD and NHHR in T2DM patients. The sample included in this study is nationally representative, and the conclusions are well generalized. In addition, the study’s results were validated through sensitivity analyses, confirming their reliability. However, there are certain limitations to this research. Firstly, as this was a retrospective study, we were unable to make causal inferences and a large prospective study should be conducted in the future to discuss causality. Second, although we have adjusted for potential confounders, there may still be confounders that affect the risk of DKD with NHHR. Finally, the inclusion population for this study was U.S. adults, so it was not possible to analyze other special populations or other races. Further research is required in the future to determine if the effect of NHHR on DKD risk can be extended to different populations.

## Conclusion

Our research identified a non-linear correlation between NHHR and DKD risk in T2DM patients based on analysis of large-scale population data. NHHR is a valuable tool for predicting the occurrence of DKD in patients with T2DM. Early monitoring of NHHR in patients with T2DM may help assess risk and predict prognosis in this patient population. Keeping the NHHR in an appropriate range is beneficial in reducing the risk of DKD, and NHHR levels can be controlled in clinical practice by lipid-lowering medications, physical activity, weight management, and smoking cessation. In addition, NHHR can be more widely used in public health screening as a low-cost and easily accessible indicator.

## Data Availability

Publicly available datasets were analyzed in this study. This data can be found at: https://www.cdc.gov/nchs/nhanes.
