# Supplementary material for: The nonlinear relationship between the ratio of non-high-density lipoprotein cholesterol to high-density lipoprotein cholesterol and the risk of diabetic kidney disease in patients with type 2 diabetes mellitus
Source: Front Med (Lausanne). 2025 Feb 19;12:1492483. doi: 10.3389/fmed.2025.1492483 (PMC11880278; doi:10.3389/fmed.2025.1492483)
Supplement: Supplementary file 1 [file Table_1.docx]

| Supplementary Material Table 1 The weighted baseline characteristics of T2DM patients from NHANES 1999-2018 | | | | |
| --- | --- | --- | --- | --- |
| **Characteristic** | **Total** | **T2DM patients without DKD** | **T2DM patients with DKD** | ***P*-value** |
| N | 3243 | 1985 | 1258 |  |
| Age, years | 58.80±0.33 | 56.28±0.41 | 63.48±0.48 | < 0.001 |
| Sex(%) |  |  |  | 0.815 |
| Male | 1710(51.50) | 1047(51.70) | 663(51.12) |  |
| Female | 1533(48.50) | 938(48.30) | 595(48.88) |  |
| Race(%) |  |  |  | 0.688 |
| Mexican American | 647( 8.85) | 393(8.46) | 254(9.58) |  |
| Non-Hispanic Black | 713(13.10) | 442(12.83) | 271(13.61) |  |
| Non-Hispanic White | 1318(65.63) | 775(66.09) | 543(64.78) |  |
| Other Hispanic | 308( 5.60) | 200(5.61) | 108(5.60) |  |
| Other Race | 257( 6.81) | 175(7.02) | 82(6.43) |  |
| Education levels(%) |  |  |  | < 0.001 |
| Below high school | 558( 9.48) | 306( 7.68) | 252(12.82) |  |
| High school | 1334(41.18) | 798(39.74) | 536(43.87) |  |
| Above high school | 1351(49.34) | 881(52.58) | 470(43.31) |  |
| Smoke(%) |  |  |  | 0.377 |
| No | 1616(49.16) | 1007(49.99) | 609(47.62) |  |
| Yes | 1627(50.84) | 978(50.01) | 649(52.38) |  |
| Alcohol use(%) |  |  |  | 0.01 |
| No | 569(15.73) | 328(14.21) | 241(18.57) |  |
| Yes | 2674(84.27) | 1657(85.79) | 1017(81.43) |  |
| Hypertension(%) |  |  |  | < 0.001 |
| No | 950(31.19) | 715(37.14) | 235(20.12) |  |
| Yes | 2293(68.81) | 1270(62.86) | 1023(79.88) |  |
| Cardiovascular disease(%) |  |  |  | < 0.001 |
| No | 2481(78.00) | 1639(83.13) | 842(68.46) |  |
| Yes | 762(22.00) | 346(16.87) | 416(31.54) |  |
| Physical activity(%) |  |  |  | < 0.001 |
| No | 1981(55.56) | 1151(51.99) | 830(62.20) |  |
| Moderate | 776(26.98) | 499(28.79) | 277(23.60) |  |
| Vigorous | 486(17.47) | 335(19.22) | 151(14.20) |  |
| Lipid-lowering drugs(%) |  |  |  | 0.032 |
| No | 1785(54.12) | 1154(55.92) | 631(50.76) |  |
| Yes | 1458(45.88) | 831(44.08) | 627(49.24) |  |
| PIR | 2.75±0.04 | 2.88±0.05 | 2.51±0.06 | < 0.001 |
| BMI, kg/m^2^ | 32.67±0.19 | 32.82±0.23 | 32.41±0.30 | 0.264 |
| HbA1c, % | 6.97±0.04 | 6.83±0.04 | 7.23±0.07 | < 0.001 |
| FPG, mg/dL | 148.77±1.36 | 145.08±1.48 | 155.64±2.26 | < 0.001 |
| ALT, IU/L | 28.17±0.39 | 29.21±0.57 | 26.24±0.57 | < 0.001 |
| AST, IU/L | 26.42±0.32 | 26.59±0.43 | 26.10±0.48 | 0.457 |
| eGFR, ml/min/1.73m^2^ | 85.01±0.52 | 92.27±0.53 | 71.51±1.02 | < 0.001 |
| Cr, mg/dL | 0.95±0.01 | 0.83±0.01 | 1.19±0.04 | < 0.001 |
| UA, mg/dL | 5.86±0.04 | 5.63±0.04 | 6.28±0.06 | < 0.001 |
| BUN, mg/dL | 15.78±0.15 | 13.95±0.15 | 19.19±0.35 | < 0.001 |
| Ualb, mg/L | 126.60±13.69 | 12.46± 0.32 | 342.14±37.41 | < 0.001 |
| Ucr, mg/dL | 124.83±1.84 | 128.12±2.42 | 118.63±2.92 | 0.016 |
| UACR, mg/g | 119.09±12.04 | 9.96± 0.18 | 325.15±32.75 | < 0.001 |
| TG, mg/dL | 173.81±4.35 | 165.78±5.02 | 188.76±7.61 | 0.01 |
| TC, mg/dL | 190.63±1.12 | 190.77±1.39 | 190.35±1.82 | 0.854 |
| HDL-C, mg/dL | 48.87±0.39 | 48.40±0.44 | 49.73±0.73 | 0.115 |
| LDL-C, mg/dL | 108.80±0.88 | 110.47±1.12 | 105.56±1.39 | 0.006 |
| NHHR | 3.19±0.04 | 3.22±0.05 | 3.16±0.05 | 0.403 |

Continuous variables were presented as Mean±SE, *P*-value was calculated by survey-weighted linear regression. Categorical variables were presented as the percentage (95% confidence interval), *P*-value was calculated by the survey-weighted Chi-square test.

NHANES, National Health and Nutrition Examination Survey; PIR, poverty income ratio; BMI, body mass index; HbA1c, glycated hemoglobin; FPG, fasting plasma glucose; ALT, alanine aminotransferase; AST, aspartate aminotransferase; eGFR, estimated glomerular filtration rate; Cr, creatinine; UA, uric acid; BUN, blood urea nitrogen; Ualb, urinary albumin; Ucr, urine creatinine; UACR, urinary albumin/creatinine ratio; TG, triglyceride; TC, total cholesterol; HDL-C, high-density lipoprotein cholesterol; LDL-C, low-density lipoprotein cholesterol; NHHR, non-high-density lipoprotein cholesterol to high-density lipoprotein cholesterol ratio.
