# Supplementary material for: The nonlinear relationship between the ratio of non-high-density lipoprotein cholesterol to high-density lipoprotein cholesterol and the risk of diabetic kidney disease in patients with type 2 diabetes mellitus
Source: Front Med (Lausanne). 2025 Feb 19;12:1492483. doi: 10.3389/fmed.2025.1492483 (PMC11880278; doi:10.3389/fmed.2025.1492483)
Supplement: Supplementary file 2 [file Table_2.docx]

| Supplementary Material Table 2 Subgroup analyses of the relationship between NHHR and DKD | | | | |
| --- | --- | --- | --- | --- |
| **OR(95%CI) *P*-value** | | | | |
|  | **NHHR≤ 2.82** | ***P* for interaction** | **NHHR＞2.82** | ***P* for interaction** |
| Age |  | 0.573 |  | < 0.001 |
| <60 | 0.76(0.48,1.20) 0.231 |  | 0.80(0.69,0.92) 0.002 |  |
| ≥60 | 0.68(0.49,0.95) 0.025 |  | 1.03(0.95,1.13) 0.448 |  |
| Sex |  | 0.522 |  | 0.707 |
| Male | 0.78(0.51,1.19) 0.238 |  | 0.97(0.87,1.07) 0.527 |  |
| Female | 0.62(0.42,0.91) 0.015 |  | 0.94(0.83,1.06) 0.300 |  |
| Race |  | 0.061 |  | 0.051 |
| Mexican American | 0.42(0.24,0.76) 0.006 |  | 0.94(0.77,1.15) 0.556 |  |
| Non-Hispanic Black | 0.56(0.31,0.98) 0.044 |  | 0.86(0.70,1.06) 0.167 |  |
| Non-Hispanic White | 0.67(0.43,0.84) 0.037 |  | 0.98(0.88,1.09) 0.708 |  |
| Other Hispanic | 0.63(0.18,2.27) 0.455 |  | 1.08(0.77,1.51) 0.665 |  |
| Other Race | 2.80(1.09,7.21) 0.034 |  | 0.82(0.58,1.16) 0.256 |  |
| HbA1c |  | 0.321 |  | 0.520 |
| <7 | 0.77(0.59,0.99) 0.044 |  | 0.92(0.83,1.02) 0.115 |  |
| ≥7 | 0.59(0.42,0.83) 0.002 |  | 0.99(0.87,1.13) 0.923 |  |
| BMI |  | 0.769 |  | 0.139 |
| <25 | 0.78(0.46,1.33) 0.365 |  | 1.02(0.89,1.18) 0.738 |  |
| ≥25,<30 | 0.72(0.49,1.07) 0.101 |  | 0.88(0.74,1.04) 0.138 |  |
| ≥30 | 0.70(0.52,0.93) 0.015 |  | 0.95(0.86,1.06) 0.371 |  |
| Smoke |  | 0.476 |  | 0.650 |
| No | 0.66(0.49,0.88) 0.005 |  | 0.96(0.86,1.07) 0.419 |  |
| Yes | 0.75(0.56,1.00) 0.051 |  | 0.94(0.84,1.05) 0.258 |  |
| Hypertension |  | 0.642 |  | 0.589 |
| No | 0.52(0.32,0.84) 0.008 |  | 0.90(0.75,1.07) 0.236 |  |
| Yes | 0.73(0.58,0.92) 0.007 |  | 0.96(0.88,1.05) 0.388 |  |
| CVD |  | 0.482 |  | 0.417 |
| No | 0.73(0.57,0.94) 0.013 |  | 0.97(0.89,1.06)0.473 |  |
| Yes | 0.58(0.39,0.86) 0.007 |  | 0.94(0.78,1.12) 0.470 |  |

adjusted for age, sex, race, PIR, education level, smoke, alcohol use, physical activity, BMI, hypertension, CVD, lipid-lowering drugs, FBG, HbA1c, ALT, AST, Cr, UA, BUN, and TG.
